# Protective Effect of an Exopolysaccharide Produced by *Lactiplantibacillus plantarum* BGAN8 Against Cadmium-Induced Toxicity in Caco-2 Cells

**DOI:** 10.3389/fmicb.2021.759378

**Published:** 2021-11-01

**Authors:** Emilija Brdarić, Svetlana Soković Bajić, Jelena Đokić, Slađana Đurđić, Patricia Ruas-Madiedo, Magdalena Stevanović, Maja Tolinački, Miroslav Dinić, Jelena Mutić, Nataša Golić, Milica Živković

**Affiliations:** ^1^Group for Probiotics and Microbiota-Host Interaction, Laboratory for Molecular Microbiology, Institute of Molecular Genetics and Genetic Engineering, University of Belgrade, Belgrade, Serbia; ^2^Faculty of Chemistry, University of Belgrade, Belgrade, Serbia; ^3^Department of Microbiology and Biochemistry of Dairy Products, Instituto de Productos Lácteos de Asturias – Consejo Superior de Investigaciones Científicas (IPLA-CSIC), Asturias, Spain; ^4^Institute of Technical Sciences, Serbian Academy of Sciences and Arts, Belgrade, Serbia

**Keywords:** cadmium, exopolysaccharides, *Lactiplantibacillus plantarum*, intestinal epithelial cells, inflammation, oxidative stress, cellular junctions

## Abstract

Cadmium (Cd) ranks seventh on the list of most significant potential threats to human health based on its suspected toxicity and the possibility of exposure to it. It has been reported that some bacterial exopolysaccharides (EPSs) have the ability to bind heavy metal ions. We therefore investigated the capacity of eight EPS-producing lactobacilli to adsorb Cd in the present study, and *Lactiplantibacillus plantarum* BGAN8 was chosen as the best candidate. In addition, we demonstrate that an EPS derived from BGAN8 (EPS-AN8) exhibits a high Cd-binding capacity and prevents Cd-mediated toxicity in intestinal epithelial Caco-2 cells. Simultaneous use of EPS-AN8 with Cd treatment prevents inflammation, disruption of tight-junction proteins, and oxidative stress. Our results indicate that the EPS in question has a strong potential to be used as a postbiotic in combatting the adverse effects of Cd. Moreover, we show that higher concentrations of EPS-AN8 can alleviate Cd-induced cell damage.

## Introduction

Cadmium (Cd) is a toxic metal and widespread environmental pollutant with serious adverse effects on human and animal health. In 1993, Cd was classified as a human carcinogen and teratogen (IARC Monographs on the Evaluation of Carcinogenic Risks to Humans, 1993). Cd intoxication has been linked with various diseases, including cancer, diabetes mellitus, cardiovascular diseases, neurodegeneration, and osteomalacia (Genchi et al., 2020). Development of industry, fume inhalation, and use of Cd in paint pigments, Cd–nickel batteries, electroplating, and fertilizers have resulted in high exposure to this metal, with a mortality rate of 17% (International Programme On Chemical Safety, 1992; Nawrot et al., 2010). In addition, its long half-life and low level of excretion make Cd an even more dangerous toxicant. Cd is readily transported from soil to plants with a high bioconcentration factor. In rice, for example, as one of the major staple cereal crops for most of the world’s population, the bioconcentration factor of Cd is from 0.300 to 1.112, and it affects the major physiological properties of the plant (Liu et al., 2015). In that context, it is important to mention the so-called “itai-itai” disease that hit Japan during the last century and was a consequence of prolonged intake of Cd-contaminated rice (Huang et al., 2009). An increase of Cd concentration leads to high contamination of the food chain, making food and drinking water the main sources of Cd exposure for the nonsmoking population (Satarug et al., 2010). The first target of orally taken Cd is preferentially the gastrointestinal tract (GIT) (Goon and Klaassen, 1989). Cadmium causes intestinal inflammation, death of epithelial cells, and morphological alterations of cell junctions, which leads to a leaky intestinal barrier (Blais et al., 1999; Prozialeck, 2000; Zhao et al., 2006; Ninkov et al., 2015). After absorption by intestinal epithelium, Cd is transported via blood circulation to different organs and tissues. Thus, keeping the intestinal barrier’s integrity intact is of crucial importance.

In view of all these facts, it is urgent to find novel strategies to prevent and neutralize the toxic effect of Cd. It has been reported that some lactic acid bacteria such as lactobacilli, which are commonly present in the GIT and have GRAS status, can bind toxic metal ions and detoxify them (Halttunen et al., 2008; Mrvcic et al., 2009). These abilities often correlate with the presence of different surface biomolecules [e.g., exopolysaccharides (EPSs)] responsible for the probiotic activity of lactobacilli (Yi et al., 2017). EPSs are carbohydrate polymers, which are covalently or loosely bound to the cell surface or secreted in the cell environment and can have antioxidative and immunomodulatory properties (Kanmani et al., 2011; Jones et al., 2014; Caggianiello et al., 2016). Besides, EPS might be used as a postbiotics (Wegh et al., 2019), which are defined as a preparation of inanimate microorganisms and/or their components that confers a health benefit on the host (Salminen et al., 2021). Adsorption of heavy metals by EPSs is mainly a metabolism-independent process based on physicochemical interactions between metal cations and negatively charged acidic functional groups of EPSs (Morillo Pérez et al., 2008). Sequestration of metals is attributable to the presence of various functional groups such as carboxyl, acetate, hydroxyl, amine, phosphate, and sulfate in extracellular bacterial polymers, and the result of metal sequestration may be physical sorption, ion exchange, complexation, and/or precipitation (Gadd and White, 1989; Liu and Fang, 2002). It is now well known that EPS molecules from different bacteria are very potent Cd binders in aqueous solution (Polak-Berecka et al., 2014), but to the best of our knowledge, evidence on how they act in other conditions is insufficient.

The aims of the present study were to select EPS-producing lactobacilli with the highest ability to adsorb Cd from our laboratory collection and show whether the action of EPSs can lead to *in vitro* prevention of Cd-induced inflammation, oxidative stress, and disruption of the cell junctions of differentiated Caco-2 cells, which are well-known test objects frequently used as *in vitro* models to estimate the ability of chemicals to cross the gut barrier and define their mechanisms in humans (Yee, 1997; Angelis and Turco, 2011). Providing information about EPS efficiency is particularly significant in view of the current trend of replacement of live bacteria with nonviable bacterial extracts and metabolic by-products in order to reduce health risks (Konstantinov et al., 2013; Patel and Denning, 2013) and/or exclude biological effects that rely on bacterial metabolism, which might be variable under different conditions (Tsilingiri et al., 2012; Tsilingiri and Rescigno, 2013).

## Materials and Methods

### Bacterial Strains, Media, and Growth Conditions

The eight EPS-producing lactobacilli used in this study are listed in Table 1. All strains were grown in De Man–Rogosa–Sharpe medium [MRS (Merck, GmbH, Darmstadt, Germany)]. We prepared MRS agar plates by adding 1.7% agar (Torlak, Belgrade, Serbia). Bacterial strains were grown under anaerobic or aerobic conditions at 30°C or 37°C, depending on the strain.

**TABLE 1:** List of strains used in this study.

Species	Strain	Origin
*Lactiplantibacillus plantarum*	BGAN8	Cow white cheese
	BGPKM22	Cow sour milk
	BGVL2a-18	Goat cheese
	BGMI1	Cow white cheese
	BGSJ2-3	Cow white cheese
*Lacticaseibacillus rhamnosus*	BGHI22	Human intestinal tract
	BGHV954	Human vaginal tract
	BGHV20	Human vaginal tract

### Exopolysaccharide Extraction and Purification

Exopolysaccharide (EPS) molecules were isolated from *Lactiplantibacillus plantarum* BGAN8 (EPS-AN8), the selected strain with the best Cd-binding ability. For EPS isolation, 100 μL of overnight BGAN8 culture was spread on 200 MRS agar plates and incubated for 48 h at 30°C. Isolation of EPSs was done according to the protocol provided by Ruas-Madiedo et al. (2006) with additional steps described by Dinić et al. (2018). Dialysis was performed twice, after the isolation and purification steps, and lasted for 5 days, each time with daily changes of Milli-Q water. The dialysis bag (Sigma–Aldrich, St. Louis, MO, United States) had a 12- to 14-kDa molecular mass cutoff. At the end of dialysis, extracted and purified EPS molecules were lyophilized (Alpha 1-4 LSC plus freeze dryer, Martin Christ, Germany).

### Analysis of EPS-AN8 Structure by Size Exclusion Chromatography–Multiangle Laser Light Scattering and Testing of Sugar Composition of the Purified Exopolysaccharide

First, the purified EPS was assessed by means of size exclusion chromatography (SEC) coupled with a multiangle laser light scattering (MALLS) detector as described by Nikolic et al. (2012). The chromatographic system (Waters, Milford, MA, United States) was composed of an Alliance 2690 module injector, a Photodiode Array PDA 996 detector (set at 280 nm), a 410 refractive index detector, and the Empower software (Waters). The MALLS detector (Dawn Heleos II, Wyatt Europe GmbH, Dambach, Germany) was coupled in series, and the Astra 3.5 software was used for analysis of molar mass distribution. Separation was carried out in two SEC columns placed in series, TSK-Gel G3000 PW_*XL*_ + TSK-Gel G5000 PW_*XL*_, protected with a TSK-Gel guard column (Supelco-Sigma), at a temperature of 40°C and flow rate of 0.45 mL/min using 0.1 M NaNO_3_ as the mobile phase. Experiments were repeated three times.

For analysis of neutral sugars in testing EPS monosaccharide composition, polysaccharides (approximately 1.6 mg) were first hydrolyzed with 3M TFA (121°C, 90 min). Monosaccharides were converted into their corresponding alditol acetate by reduction with NaBH_4_ and subsequent acetylation (Laine et al., 1972). Identification and quantification were performed by gas–liquid chromatography (GLC) on a gas chromatograph equipped with a DB-5HT column (Agilent, Santa Clara, CA, United States; 30 m × 0.25-mm internal diameter; 0.10-mm film thickness) coupled to a quadrupole mass detector. The oven program started at 175°C for 1 min and was increased by 2.5°C min/min until 204°C was reached. Helium was used as the carrier gas at a flow rate of 1 mL/min. Identification was performed on the basis of coincidence of the retention time of sample components with those previously measured for standards analyzed in identical conditions, using inositol as an internal standard. GLC was performed in the Centro de Investigaciones Biológicas (CIB) by Margarita Salas, CSIC, 28040 Madrid, Spain.

### Fourier Transform Infrared Spectroscopy

As a powerful analytical technique to investigate the structural characteristics of biomacromolecules (Verhoef et al., 2005), Fourier transform infrared spectroscopy (FTIR) spectroscopy was used to confirm the qualitative composition of EPS-AN8 molecules. The FTIR spectrum of samples was acquired in the transmittance mode on a Nicolet iS10 spectrometer (Thermo Fisher Scientific, Waltham, MA, United States) to confirm the qualitative composition of samples, that is, to confirm that extracted and purified material from BGAN8 is an EPS. Measurements were performed in the spectral range of 400 to 4,000 cm^–1^ with a resolution of 4 cm^–1^, the number of scans being 32. Spectra were collected using the attenuated total reflectance mode, whereas the OMNIC software was used to acquire, process, analyze, and manage FTIR data in a graphical environment.

### Preparation of Cadmium Solutions

Cadmium (Cd) was added in the form of CdCl_2_ (Sigma–Aldrich). It was dissolved in Milli-Q water at a concentration of 1 mM and kept at 4°C. Working solutions were freshly made by dissolving CdCl_2_ in Milli-Q water or cell culture medium.

### Cadmium-Binding Experiments

The ability of live EPS-producing lactobacilli to bind Cd was investigated using the protocol provided by Zhai et al. (2013) with slight modifications. Overnight cultures were washed twice in phosphate-buffered saline (PBS). A volume of 1 mL (10^9^ CFU/mL) was taken and centrifuged at 13 000 revolutions/min (rpm) for 10 min at room temperature (RT). Cell pellets were resuspended in 1 mL of dissolved CdCl_2_ (50 μM) and incubated at 30°C for 1 h. After 1 h, the mixtures were centrifuged (13 000 rpm, 20 min, RT), and supernatants were collected and kept at −20°C for residual Cd content analysis. The concentration of 50 μM CdCl_2_ used in this protocol corresponded to the doses of Cd present in the environment (Blanusa et al., 2002; Damek-Poprawa and Sawicka-Kapusta, 2004).

The EPS of a strain with the highest ability to adsorb Cd ions was isolated, and its adsorption of Cd was measured, following the protocol of Polak-Berecka et al. (2014) with a few modifications. Briefly, 1 mg/mL of EPS was resuspended in Milli-Q water and placed in a dialysis bag. The EPS-containing dialysis bag was placed in a glass cup filled with an aqueous solution of CdCl_2_ and stirred for 24 h at 30°C.

### Determination of Cadmium Concentration

Concentrations of Cd ions were determined by inductively coupled plasma mass spectrometry with ICP-QMS (iCAP Q, Thermo Scientific X series 2, United Kingdom). A standard stock solution of Cd containing of 1,000.0 ± 0.2 mg/L (Alfa Aesar, Germany) was used to prepare intermediate standard solutions for ICP-MS measurements. Operating conditions for ICP-QMS were as follows: RF power—1548 W; lens voltage—7 V; pulse stage voltage—950 V; sample uptake rate—24 rpm; gas flow rates—13.9, 1.09, and 0.8 L/min; acquisition time—3 × 50 s; points per peak - 3; dwell time—10 ns; detector mode—analog/pulse; replicates—3; measured isotope—^113^Cd.

### Cell Culture

Differentiated human enterocyte-like Caco-2 cells were used as a model system to analyze the adverse effects of Cd and putative protection by the EPS. The Caco-2 cells were grown and maintained in the same manner as described by Popović et al. (2019). The medium was replaced every second day for 21 days.

### Treatment of Caco-2 Cells

For all assays, cells were differentiated in 24-well plates, except in the case of the Lucifer yellow test, where cells were differentiated in 24-well plates covered with Transwell inserts (pore diameter 0.4 μm, Sarstedt, Nümbrecht, Germany). Here 21-day cells were washed three times with PBS and then treated. First, in order to find subtoxic concentrations of CdCl_2_, cells were treated with CdCl_2_ in a range of different concentrations (50, 100, and 200 μM) for 24 h. EPS-AN8 was added in two concentrations: in a lower concentration (50 μg/mL), which corresponds to approximately 1 × 10^9^ CFU/mL *L. plantarum* BGAN8 and in a two times higher concentration (100 μg/mL). Both were in a range of concentrations usually used in different studies (Liu et al., 2019). Besides, none of used EPS-AN8 concentrations caused cytotoxic effect on Caco-2 (Supplementary Data). Further on, Caco-2 cells were simultaneously treated with EPS-AN8 and with a subtoxic concentration of CdCl_2_ for 3 h, which was enough time to observe Cd-induced changes in gene expression (Rusanov et al., 2015) and 24 h to measure protein expression (Zhai et al., 2016). At the end of the treatment, cell culture supernatants were collected and stored at -20°C for cytotoxicity assay and superoxide dismutase (SOD) activity measurements. Cells were detached with trypsin-EDTA solution (Torlak) and stored at -80°C for quantitative real-time polymerase chain reaction (PCR) and Western blot analysis. Also, to see if the EPS could be used to alleviate the effect of CdCl_2_ on the cell, Caco-2 was first treated with a subtoxic concentration of CdCl_2_ for 24 h. The next day the medium was changed, the EPS was added in lower and higher concentrations, and the incubation lasted another 24 h.

### Cytotoxicity Assay

The level of lactate dehydrogenase (LDH) released in supernatants, which correlates with the number of dead cells (Bajić et al., 2020b), was measured by using an LDH cytotoxicity assay kit (Thermo Fisher Scientific, Waltham, MA, United States). Activity of LDH was measured in cell culture supernatants, following the manufacturer’s instructions. Absorbance was read at 450 nm using a microplate reader (Tecan Austria GmbH, Grödig, Austria).

### Quantitative Real-Time Polymerase Chain Reaction

Total RNA was isolated following the protocol described by Sokovic Bajic et al. (2019). For reverse transcription, the RevertAid RT kit was used according to the manufacturer’s protocol (Thermo Fisher Scientific). Amplification of synthesized cDNA was done in a 7500 real-time PCR system (Applied Biosystems, Foster City, CA, United States). The SYBR^TM^ Green PCR Master Mix (Applied Biosystems) was used following the instructions for reaction conditions, viz.: activation at 95°C for 10 min, 40 cycles of 15 s at 95°C, and 60 s at 60°C. The primers used are listed in Table 2. Expression of mRNA was normalized against the *GAPDH* gene utilizing the 2^–ΔΔ*Ct*^ method. All primers were purchased from Thermo Fisher Scientific.

**TABLE 2:** List of primers used for real-time PCR analysis in this study.

Primer name	Primer sequence	References
IL-8	5′-ACACAGAGCTGCAGAAATCAGG-3′	Angrisano et al., 2010
	5′-GGCACAAACTTTCAGAGACAG-3′	
CDH1	5′-AGCCTGTCGAAGCAGGATTG-3′	Popović et al., 2020
	5′-AGAAACAGCAAGAGCAGCAGA-3′	
OCLN	5′-TCAGGGAATATCCACCTATCACTTCAG-3′	Elamin et al., 2012
	5′-CATCAGCAGCAGCCATGTACTCTTCAC-3′	
ZO-1	5′-AGGGGCAGTGGTGGTTTTCTGTTCTTTC-3′	Elamin et al., 2012
	5′-GCAGAGGTCAAAGTTCAAGGCTCAAGAGG-3′	
NQO1	5′-GGATTGGACCGAGCTGGAAA-3′	This work
	5′-CAAACTGAAACACCCAGCCG-3′	
GAPDH	5′-GTGAAGGTCGGAGTCAACG-3′	Freudenberger et al., 2014
	5′-TGAGGTCAATGAAGGGGTC-3′	

### Western Blot

Protein isolation and Western blotting were performed following the protocol provided by Popović et al. (2019). Isolated proteins were separated on 12% sodium dodecyl sulfate–polyacrylamide gel electrophoresis and transferred to a 0.2-mm nitrocellulose membrane (GE Healthcare). Anti-GAPDH (as loading control; 1:1,000; Invitrogen), anticlaudin (CLDN-4, 1:1,000; Thermo Fisher Scientific), and anti-p65 [nuclear factor κB (NF-κB), 1:500; Cell Signaling Technology] were used during the night at +4°C. Proteins were detected by a ChemiDoc apparatus and quantified in ImageJ software (National Institutes of Health).

### Permeability Assay

Integrity of the monolayer barrier was determined by measuring the passive passage of Lucifer yellow dye (Invitrogen, Thermo Fisher Scientific). The protocol described by Yamaura et al. (2016), with a few modifications, was followed. Initially, Lucifer yellow was dissolved in dimethyl sulfoxide and then diluted 1,000-fold in PBS solution. The mixture was added to the apical membrane of Caco-2 cells and incubated for 1 h at 37°C. A microplate reader (Tecan) with excitation and emission of 428 and 536 nm, respectively, was used for fluorescence detection.

### Superoxide Dismutase Activity

Superoxide dismutase (SOD) activity was measured in supernatants of Caco-2 cells using the SOD Assay Kit (Sigma–Aldrich) after 24 h of treatments. The manufacturer’s instructions were followed, and absorbance was measured at 440 nm, using a microplate reader (Tecan).

### Statistical Analysis

GraphPad Prism 8 software was used in performing statistical analysis and preparing graphs. All experiments were repeated at least three times. After checking normal distribution of values with the Kolmogorov–Smirnov test, one-way analysis of variance and Dunnett and Tukey tests were used for multiple comparison. Values that do not share a common letter are significantly different at *p* < 0.05. Data are presented as mean values ± standard deviation from different experiments.

## Results and Discussion

### Ability of Exopolysaccharide-Producing Lactobacilli to Adsorb Cd Ions and Characterization of EPS-AN8

**FIGURE 1:**
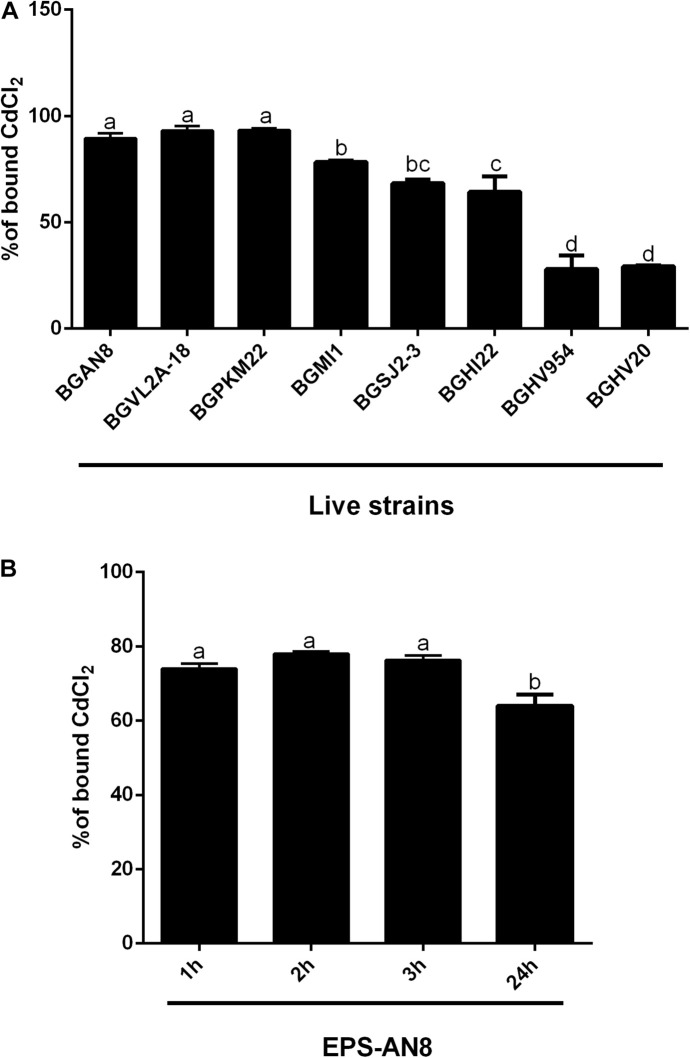
Ability of live exopolysaccharide (EPS)–producing lactobacillus strains to bind Cd **(A)** and ability of EPS isolated from the selected *Lactiplantibacillus plantarum* AN8 strain (EPS-AN8) to adsorb Cd ions after 1, 2, 3, and 24 h of exposure **(B)**. Values that do not share a common letter are significantly (*p* < 0.05) different.

**FIGURE 2:**
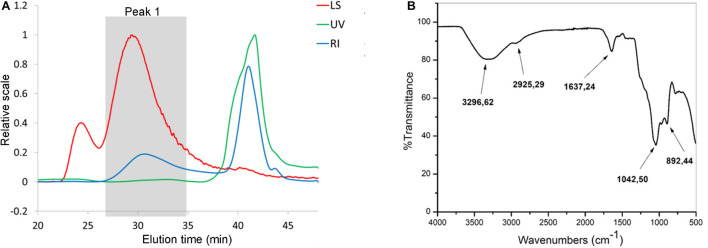
SEC analysis **(A)** of EPS-AN8. Ultraviolet (UV, 280 nm) detector, multiangle light scattering (LS, angle 90°), refraction index (RI). **(B)** FTIR spectra of EPS-AN8.

It is known that microorganisms can interact with heavy metals via biosorption of metal ions on the cell surface, intracellular uptake, and chemical transformation (Hassan et al., 2009). Also, it was previously reported that the greatest amount of metals is bound to the surface and adsorbed, in contrast to an exceedingly small part of ions that was actively taken up (Mrvcic et al., 2009). Lactobacilli turned out to be a promising solution for the growing problem of Cd pollution (Mrvčić et al., 2012). They can adsorb a great number of different metals ions, in a strain-specific manner (Zhai et al., 2013). For example, lactobacilli showed a protective effect against acute Cd poisoning in mice. More precisely, both live and dead lactobacilli decreased intestinal absorption of Cd ions, leading to a lower level of Cd in tissues and increased concentration in feces, which indicates the involvement of surface biomolecules of lactobacilli in the Cd-binding process (Zhai et al., 2013). Lactobacilli have the ability to produce homopolysaccharides and heteropolysaccharides with enormous structural diversity and different biological properties (Caggianiello et al., 2016). Among other activities, the potential of different EPS-producing bacterial species to adsorb metal ions was described by Yi et al. (2017). Therefore, in the present study, we tested the potential of eight EPS-producing lactobacilli strains from our laboratory collection to adsorb Cd. *L. plantarum* BGAN8, *L. plantarum* BGVL2A-18, and *L. plantarum* BGPKM22 showed the highest ability to bind Cd ions in aqueous solution and did not significantly differ from each other (Figure 1A). *L. plantarum* BGAN8 has been well characterized as an EPS producer and microvesicle releaser (Bajic et al., 2020a), and it was therefore chosen for further work. Further, isolated EPS-AN8 was tested for binding Cd. As shown in Figure 1B, EPS-AN8 has a great capacity for binding Cd ions. After the first, second, and third hour of EPS and CdCl_2_ coincubation, the percentage of Cd binding did not differ significantly, ranging between 72 and 78% of the total amount of CdCl_2_ in solution. Interestingly, a slight decrease in adsorbed Cd ions was observed after 24 h of coincubation, suggesting that a smaller amount of Cd is reversibly bound to EPS molecules. This observation is in agreement with earlier studies that showed the same phenomenon of a short-term reduction of Cd ion binding between 24 and 48 h (Polak-Berecka et al., 2014). Examination of the SEC profile reveals the presence of one major peak with average retention time of about 32 min (Figure 2A). Furthermore, SEC- MALLS analyses showed that EPS-AN8 has a high molecular weight (Table 3), the value of which is within the expected range of weight among characterized HePSs of other lactobacilli from the *L. plantarum* group, ranging from 10^4^ to 10^6^ Da (Tallon et al., 2003; Sánchez et al., 2006; Nikolic et al., 2012). Also, radius of gyration was similar to values for EPS of other lactobacilli (Bachtarzi et al., 2020). Analysis of neutral sugar content of EPS-AN8 revealed that glucose, galactose, rhamnose, and mannose are present in a ratio of 1:0.33:0.19:0.05, respectively, and in percentages of 12.91%, 4.23%, 2.45%, and 0.60% (Table 3). It is well known that heteropolysaccharides consist of different monosaccharides, such as D-glucose, D-galactose, and L-rhamnose (De Vuyst and Degeest, 1999). The presence of mannose was also reported in other HePSs as well, such as in *Lactococcus lactis* subspecies *cremoris* Ropy352, *Lactobacillus pentosus* LPS26, or *Lactobacillus paraplantarum* BGCG11 (Cerning et al., 1994; Knoshaug et al., 2000; Sánchez et al., 2006). The FTIR spectrum of EPS-AN8 is presented on Figure 2B. The spectrum contains typical groups for polysaccharides, that is, carboxyl, hydroxyl, and amide groups (Dinić et al., 2018). A broad-stretching absorption band at 3,296.62 cm^–1^ corresponds to –OH or –NH vibrations (Coates, 2006). From the literature, it is well known that hydroxyl groups are ubiquitous in polysaccharide structure, which exhibits an intense broad stretching vibration in the region characteristic of the carbohydrate ring (Dinić et al., 2018). The affinity of polysaccharides for water molecules depends on the presence of these multi-OH groups (Hu et al., 2017). The small absorption band at 2,925.29 cm^–1^ corresponds to the C-H stretching vibrations of methyl or methylene groups, regularly present in hexoses such as glucose or galactose and in deoxyhexoses such as rhamnose or fucose (Ismail and Nampoothiri, 2010). The band at 1,637.24 cm^–1^ represents vibration of the C = O stretch of the amide I band or carboxyl group (Shen et al., 2013). This may indicate the presence in the examined EPS of acidic sugars (monosaccharides with a carboxyl group at one end or both ends of their chain), which are important in view of the heavy metal–binding properties of this polymer (Angyal, 1989). Also, carboxyl and hydroxyl groups are important for the coordination responsible for stability of the EPS-metal bond (Moppert et al., 2009). The band at 1,041.50 cm^–1^ corresponds to a C-O stretch vibration or a phosphorus out-of-phase P-O-C stretch (Corbrjdge, 2007). EPSs are known to be composed of carbohydrates (sugar residues) substituted with proteins, DNA, phospholipids, and noncarbohydrate substituents such as acetate, glycerol, pyruvate, sulfate, carboxylate, succinate, and phosphate (Angelin and Kavitha, 2020). The strongest absorption band at 1,041.50 cm^–1^ indicates that the substance is an EPS. A possible explanation for the effective binding of metal cations is that the phosphate group undergoes deprotonation under physiological conditions, which results in negative charges along the phosphate backbone (Pal and Paul, 2008; Ruan et al., 2008). The resulting negative charges tend to be stabilized and neutralized by the binding of metal cations, such as, in our case, Cd, and result in immobilization of the metal. The absorption band at 892.44 cm^–1^ corresponds to vibrations of the glycoside link C-O-C (Nikonenko et al., 2000). Apart from the aforementioned peaks, there are no other peaks that can be observed in the spectrum of our sample.

**TABLE 3:** Physicochemical characteristics of EPS-AN8.

Parameters		
Molecular weight (g/mol = Da)	2.27 ± 0.07 × 10^5^	
Radius of gyration (nm)	86.65 ± 3.46	

**Monosaccharides**	**%**	**Ratio**

Glucose	12.91	21.5
Galactose	4.23	7.1
Rhamnose	2.45	4.1
Manose	0.60	1

### Protective Effect of EPS-AN8 on Cd-Induced Inflammation of Caco-2 Cells

**FIGURE 3:**
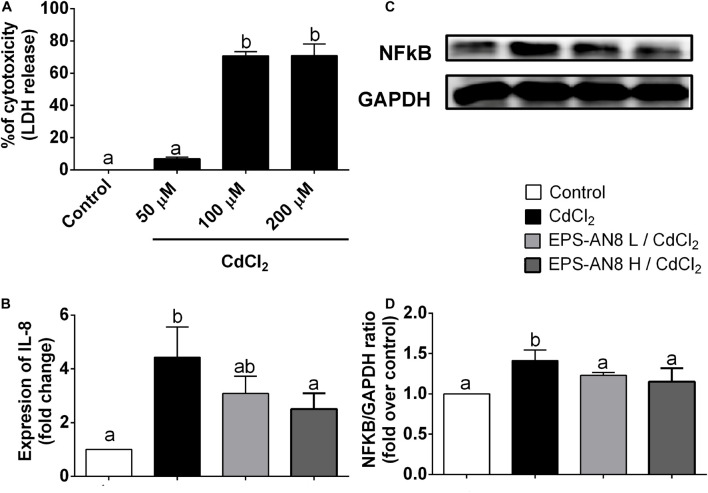
Protection from Cd-induced inflammation. **(A)** Percentage (%) of cytotoxicity measured in differentiated Caco-2 cells treated with different concentrations (50, 100, and 200 μM) of CdCl_2_. **(B)** Effect of Cd and cotreatment with Cd and EPS-AN8 in lower and higher concentrations (L and H) on *IL-8* mRNA expression after 3 h. **(C,D)** P65 protein levels after 24 h of incubation and Western blot and densitometric analysis, respectively. Values that do not share a common letter are significantly (*p* < 0.05) different.

In order to investigate the ability of EPS-AN8 to protect differentiated Caco-2 cells from Cd-induced inflammation, we determined the subtoxic concentration of CdCl_2_ on Caco-2 cells (Figure 3A), which were used as an *in vitro* model of the intestinal epithelium. Concentrations of CdCl_2_ higher than 50 μM were toxic, which is in accordance with published data (Hyun et al., 2007), so 50 μM of CdCl_2_ was used as a subtoxic concentration in our further experiments. Intestinal epithelial cells are very important, not only as a physical barrier, but also as a part of innate mucosal immunity–producing antimicrobial molecules, as well as the cytokines and chemokines required for immune response activation (Stadnyk, 1994; Turner, 2009). It has been shown that Cd induces inflammation via overproduction of interleukin 8 (IL-8) (Hyun et al., 2007). IL-8 is a chemotactic cytokine that attracts and activates leukocytes, leading to acute inflammation, which is very important for the resolution of infection (Baggiolini and Clark-Lewis, 1992). On the other hand, in the case of stimuli that cannot be removed and persist as a stimulator of the epithelial barrier and immune response, overexpression of IL-8 has a destructive effect on the local tissue (Baggiolini and Clark-Lewis, 1992). In the present study, we demonstrate that treatment of Caco-2 cells with Cd for 3 h leads to a statistically significant increase in *IL-8* gene expression (Figure 3B), but cotreatment, at the same time, with the higher concentration of EPS-AN8 provided protection from this overexpression, and the level of mRNA for IL-8 did not differ statistically from the level in the control cells (Figure 3B). On the contrary, cotreatment with the lower EPS-AN8 concentration did not affect the up-regulation of IL-8 induced by Cd. NF-κB is defined as one of the most significant regulators of inflammation in different types of cells, including intestinal epithelial cells (Mitchell et al., 2016). In addition, there are binding regions in the promoter of the IL-8 coding gene, confirming the regulatory role of NF-κB in IL-8 production (Wu et al., 1997). Importantly, it has been shown that activation of NF-κB is essential for up-regulation of IL-8 in Caco-2 cells treated with Cd (Hyun et al., 2007). Accordingly, we investigated the effect of Caco-2 cotreatment with Cd and EPS-AN8 on activation of NF-κB by estimating protein levels of phosphorylated p65, which represents a transcriptionally active form of NF-κB. As shown on Figures 3C,D, both concentrations of EPS-AN8 neutralized the stimulatory effect of Cd on NF-κB activation, but only the higher concentration of EPS-AN8 reduced IL-8m RNA levels. These results suggest that EPS-AN8, when applied simultaneously with Cd, most likely decreases Cd-induced inflammation through the sequestration of Cd ions by EPS-AN8, which inhibits entry of Cd into the cells, and imply that with the lower EPS-AN8 concentration, it is not sufficient to bind all available Cd ions responsible for mild stimulation of IL-8.

### Protective Effect of EPS-AN8 on Cd-Induced Disruption of Intercellular Junctions

**FIGURE 4:**
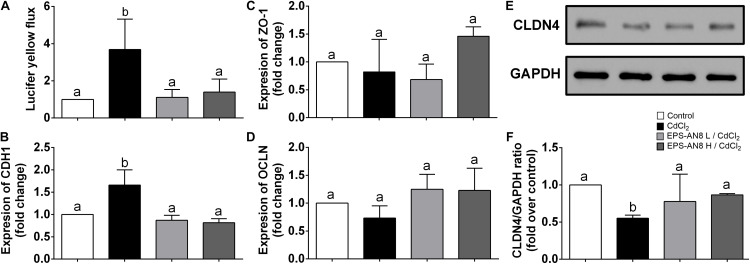
Protection from Cd-induced disruption of cell junctions by EPS-AN8. **(A)** Rate of passive transport of Lucifer yellow through Caco-2 cell monolayer after Cd exposure and coexposure with Cd and EPS-AN8 (L and H). **(B)** Effect on mRNA of adherence junction protein *CDH1* after 3 h. Effect on mRNA of tight junction proteins *ZO-1* **(C)** and *OCLN* **(D)** after 3 h. **(E,F)** Effect on expression of CLDN4 protein and representative Western blot and densitometric analysis after 24 h of incubation, respectively. Values that do not share a common letter are significantly (*p* < 0.05) different.

Maintenance of the intestinal barrier is very important in the restriction of Cd spreading in the organism (Tinkov et al., 2018). For that reason, the permeability of a differentiated layer of Caco-2 cells was analyzed after exposure of cells to Cd and to Cd and EPS simultaneously. According to the results presented in Figure 4A, Cd strongly increased permeability of the monolayer, but integrity of the monolayer was preserved in the culture cotreated with EPS-AN8. Oral administration of Cd disrupts adherence and tight junctions (TJs) in epithelial surfaces, resulting in amplified Cd absorption (Rusanov et al., 2015). Hence, the primary therapeutic targets are proteins involved in intercellular junctions. E-cadherin is defined as the most sensitive to Cd exposure, as Ca ions are substituted with ions of Cd at the sites of its binding to the cells (Prozialeck, 2000). In connection with this, we investigated the potential of two concentrations of EPS-AN8, simultaneously added to Cd, as a treatment of differentiated Caco-2 cells to neutralize the harmful effects of Cd. After 3 h of treatment, only Cd exposure significantly increased the expression of E-cadherin (*CDH1*) mRNA (Figure 4B), whereas coexposure to both concentrations of EPS-AN8 molecules retained the control value of expression. These results can be explained by the aforementioned Ca–Cd substitution and sensitivity of this protein to an increased concentration of divalent ions. Further, claudin (*CLDN-4*), zonulin-1 (*ZO-1*), and occludin (*OCLN*) are crucial proteins in forming TJs (Günzel and Fromm, 2012). In our study, the level of mRNAs for *ZO-1* and *OCLN* did not change in response to either treatment compared to the control (Figures 4C,D). In contrast, after 24-h treatment, Cd down-regulated the level of CLDN-4 (Figure 4C), whereas cotreatment with EPS-AN8 in both concentrations inhibited the downregulation of this protein’s expression (Figures 4E,F). In light of these results, it can be concluded that EPS-AN8 prevents Cd-induced destruction of the intestinal barrier by protecting against CLDN-4 degradation.

### Protective Effect of EPS-AN8 on Cd-Induced Oxidative Stress

**FIGURE 5:**
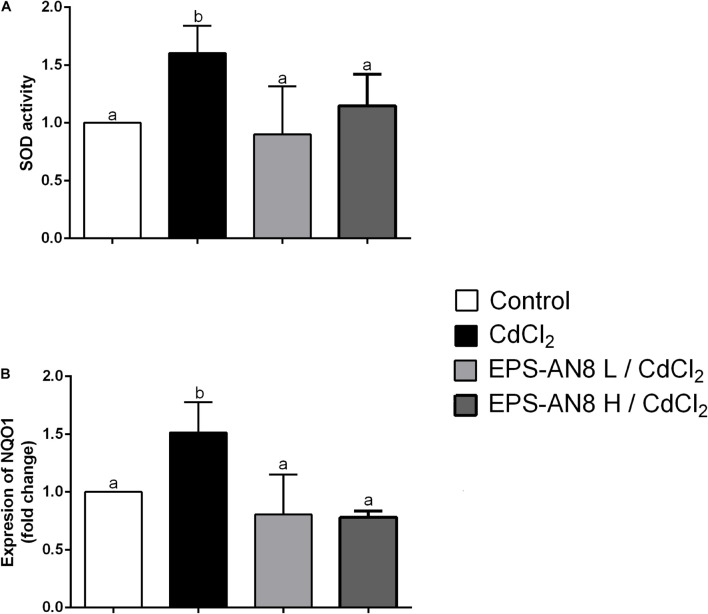
Role of EPS (L and H) in protection of Cd-induced oxidative stress. **(A)** Effect on SOD activity and **(B)** mRNA expression of *NQO1*. Values that do not share a common letter are significantly (*p* < 0.05) different.

It has been repeatedly shown that the main mechanism of Cd-induced toxicity is based on induction of oxidative stress in exposed cells (Genchi et al., 2020). We therefore investigated the activity of two enzymes important for oxidative/antioxidative responses of cells: SOD and NAD(P)H quinone reductase (NQO1). Superoxide dismutase is one of the enzymes essential for the antioxidative balance of cells, catalyzing dismutation of the superoxide anion into hydrogen peroxide and molecular oxygen (Canada and Calabrese, 1989). We were able to demonstrate that after 24 h of treatment of Caco-2 with Cd, SOD activity was significantly higher than in the control and in the case where EPS-AN8 was applied to the cells at the same time with Cd. Both concentrations of EPS were shown to be similarly efficient in inhibiting the induction of oxidative stress by Cd through activation of SOD in Caco-2 cells (Figure 5A). As SOD protects cells from the activity of reactive oxygen species (ROS), higher activity implies higher production of ROS (He et al., 2017; Wang et al., 2018). Based on this, it can be concluded that cells are in a better oxidative state in the presence of EPS-AN8 simultaneously with Cd. In addition, NQO1 is also a key enzyme of antioxidant cell defense, one that catalyzes reduction of quinones and a variety of other substrates (Pey et al., 2019). As in the case of SOD, Cd stimulated the level of mRNA for NQO1, which is consistent with data from the literature (Rusanov et al., 2015). The addition of EPS-AN8 in both lower and higher concentrations at the same time with it protected from this effect of Cd as well (Figure 5B).

### EPS-AN8 as a Potential Agent for Alleviation of Cd-Induced Damage

**FIGURE 6:**
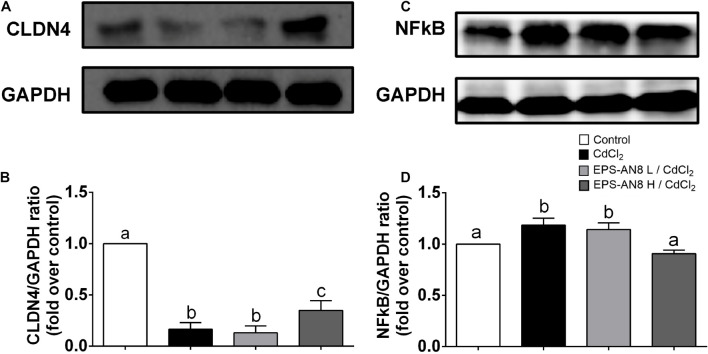
EPS-AN8 as a potential agent for alleviation of Cd-induced damage. **(A,B)** Effect of EPS (L and H) on Cd-induced down-regulation of tight junction protein CLDN-4 and representative Western blot and densitometric analysis, respectively. **(C,D)** Effect of EPS (L and H) on Cd-induced overexpression of NF-κB and representative Western blot and densitometric analysis, respectively. Values that do not share a common letter are significantly (*p* < 0.05) different.

From the results obtained when Caco-2 cells were exposed to Cd and EPS-AN8 at the same time, we conclude that EPS-AN8, in both the lower and higher tested concentrations, provides strong protection against the damage caused by Cd toxicity. We presume that such a protective effect is a consequence of the ability of EPS-AN8 to sequester Cd ions and disable the entry of ions into the cells. It was reported elsewhere that *L. plantarum* CCFM8610, besides initially sequestrating Cd ions, can also reverse damage induced by Cd in HT29 cells and mice (Zhai et al., 2016). To see if EPS-AN8 has a therapeutic effect as well, we analyzed changes in the expression of CLDN-4 and NF-κB when Caco-2 cells were previously exposed to Cd. To be specific, after 24 h of treatment of Caco-2 cells with Cd, EPS-AN8 was added to the culture as a putative agent for alleviation of Cd-induced damage and incubated for the next 24 h. Although we noticed some positive changes after treatment with the lower concentration of EPS-AN8, the higher concentration of EPS-AN8 was far more effective in this study (Figure 6). The higher concentration of EPS-AN8 significantly increased expression of CLDN-4 compared to Cd treatment (Figures 6A,B) and significantly decreased the level of p65 protein expression in comparison with Cd treatment and treatment with a lower concentration of EPS (Figures 6C,D). These results imply that EPS-AN8, besides sequestration of Cd ions, also triggered intestinal cell responses that lead to alleviation of Cd-induced damage. This assumption is in accordance with results of a previous study that demonstrated that EPS molecules might have a strong anti-inflammatory potential in conditions of high inflammation via modulation of Toll-like receptor expression and inhibition of mitogen-activated protein kinase and NF-κB in intestinal epithelial cells (Gao et al., 2017). Also, some EPSs act as a stabilizer of intestinal barrier integrity by activating signal transducers, activating transcription of signaling (STAT-3) pathways, and up-regulating TJ proteins, respectively, in cases of intestinal barrier dysfunction (e.g., inflammatory bowel disease, colitis) (Zhou et al., 2018).

## Conclusion

Our findings indicate an important role of probiotic-derived EPSs in protection against the hazardous effects of Cd on intestinal epithelial cells. To our knowledge, the present study is the first one providing information about EPSs as a potent postbiotic agent against this environmental pollutant and its possible use as a functional food supplement or dairy food additive in areas highly exposed to Cd. This pioneering work calls for further studies analyzing the use of EPSs as a putative therapeutic strategy.

## Data Availability Statement

The original contributions presented in the study are included in the article/Supplementary Material, further inquiries can be directed to the corresponding author.

## Author Contributions

EB performed all the work, analyzed and interpreted the data, and drafted this manuscript. MŽ, JĐ, and MT planned, designed, and performed some of the experiments and critically revised the manuscript. SSB performed Western blot analysis, participated in EPS extraction, analyzed the data, and interpreted the results. PR-M performed SEC-MALLS analysis, analyzed data and interpreted the results. MS analyzed FTIR spectrum of EPS-AN8 and interpreted the results. MD participated in Caco-2 cell culture experiments and qPCR. JM and SĐ performed Cd-binding research. MŽ and NG supervised all the work. All authors contributed to the article and approved the submitted version.

## Conflict of Interest

The authors declare that the research was conducted in the absence of any commercial or financial relationships that could be construed as a potential conflict of interest.

## Publisher’s Note

All claims expressed in this article are solely those of the authors and do not necessarily represent those of their affiliated organizations, or those of the publisher, the editors and the reviewers. Any product that may be evaluated in this article, or claim that may be made by its manufacturer, is not guaranteed or endorsed by the publisher.
