# Supplementary figures and images for: Protective Effect of an Exopolysaccharide Produced by Lactiplantibacillus plantarum BGAN8 Against Cadmium-Induced Toxicity in Caco-2 Cells
Source: Front Microbiol. 2021 Nov 1;12:759378. doi: 10.3389/fmicb.2021.759378 (PMC8591446; doi:10.3389/fmicb.2021.759378)

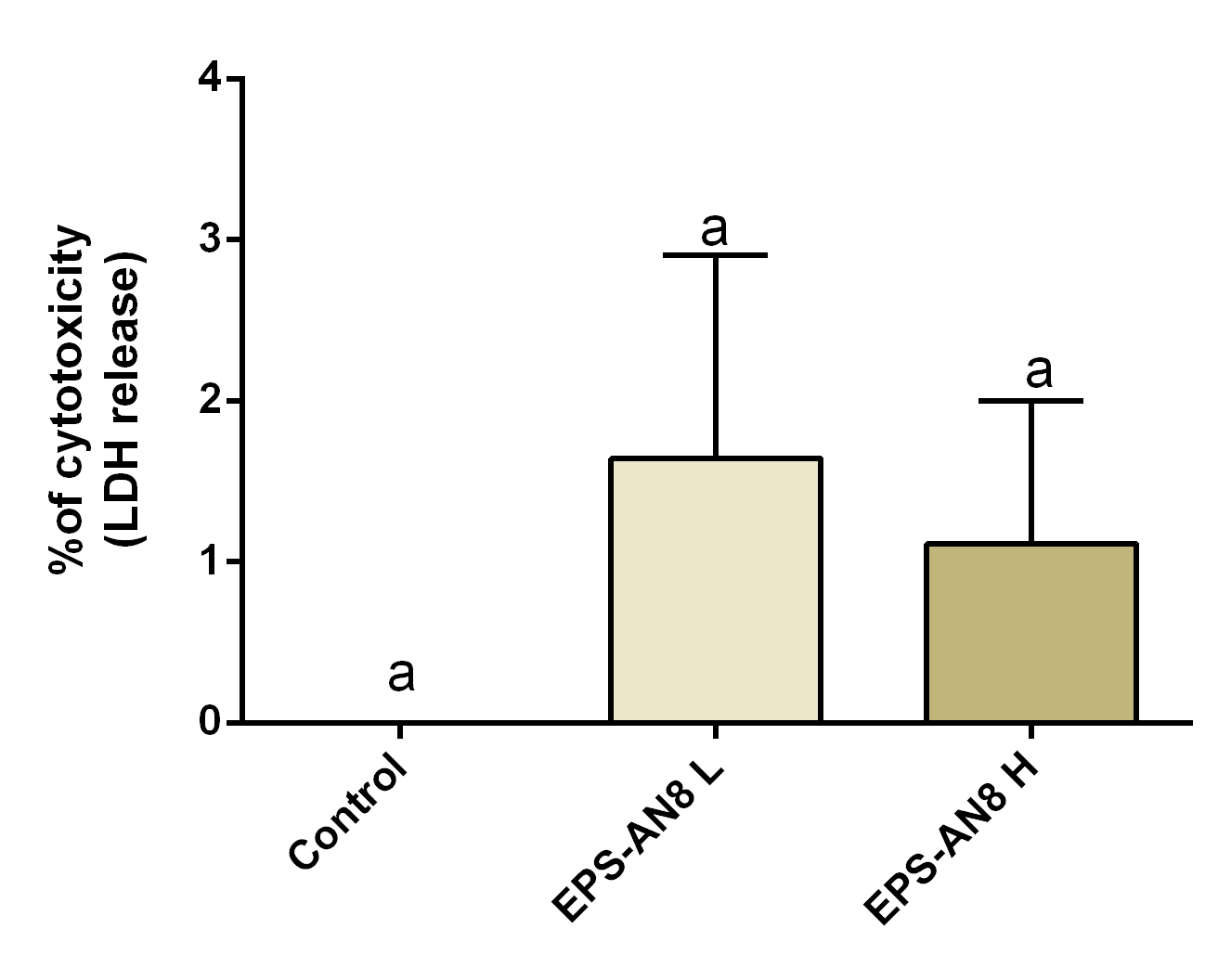

Supplement: Supplementary file 1 [file Image_1.TIF]
