# Supplementary material for: Protective Effect of an Exopolysaccharide Produced by Lactiplantibacillus plantarum BGAN8 Against Cadmium-Induced Toxicity in Caco-2 Cells
Source: Front Microbiol. 2021 Nov 1;12:759378. doi: 10.3389/fmicb.2021.759378 (PMC8591446; doi:10.3389/fmicb.2021.759378)
Supplement: Supplementary file 2 [file Table_1.DOCX]

**Supplementary figure 1.** Effect of lower (EPS-AN8 L) and higher (EPS-AN8 H) concentration EPS-AN8 on LDH release. Values that do not share a common letter are significantly (p<0,05) different.
